# ROLE OF DIFFERENT HORMONES IN THE PATHOGENESIS AND SEVERITY OF ADOLESCENT IDIOPATHIC SCOLIOSIS

**DOI:** 10.1590/1413-785220172501168600

**Published:** 2017

**Authors:** RICARDO TEIXEIRA E SILVA, RENAN JOSE RODRIGUES FERNANDES, ALLAN HIROSHI DE ARAÚJO ONO, RAPHAEL MARTUS MARCON, ALEXANDRE FOGAÇA CRISTANTE, TARCISIO ELOY PESSOA DE BARROS

**Affiliations:** 1. Universidade de São Paulo, Faculdade de Medicina, Hospital das Clínicas, Instituto de Ortopedia e Traumatologia, São Paulo, SP, Brazil; 2. Universidade de São Paulo, Faculdade de Medicina, Department of Orthopedics and Traumatology, São Paulo, SP, Brazil

**Keywords:** Scoliosis, Adolescent, Gastrin, Cortisol, Vitamin D

## Abstract

**Objective:**

To evaluate the hormonal profile of patients with adolescent idiopathic scoliosis (AIS) and its relationship to the severity of the curvature and quality of life***.***

**Method:**

Patients with scoliosis (Cobb angle above 10°), of both genders, diagnosed after 10 years of age were included, excluding those who presented other condition that could lead to scoliosis. Serum levels of 25-hydroxyvitamin D (25-OHD), cortisol and gastrin were correlated with Cobb angle and quality of life, measured by the SRS-30 questionnaire***.***

**Results:**

The levels of 25-OHD decreased in 97% of patients. There was an inverse relationship between gastrin levels and quality of life (p = 0.016). Moreover, there was an inverse correlation between the value of Cobb angle and quality of life (p = 0.036). There were no changes in cortisol levels. There was no correlation between Cobb angle and any of the hormones measured***.***

**Conclusion:**

The patients had levels of 25-OHD diminished, strengthening the hypothesis of its involvement in the development of AIS. This study also suggests that increased gastrin levels may be associated with a worse quality of life in patients with AIS. ***Level of Evidence II, Diagnostic Study.***

## RESUMO

**Objetivo:**

***Avaliar o perfil hormonal dos pacientes com escoliose idiopática do adolescente (EIA) e sua relação com a gravidade da curvatura e qualidade de vida.***

**Método:**

***Foram incluídos pacientes com escoliose (ângulo de Cobb acima de 10°), de ambos os sexos, diagnosticados após 10 anos de idade e foram excluídos aqueles que apresentassem outra condição que pudesse acarretar em escoliose. Os valores séricos da 25-hidroxivitamina D (25-OHD), cortisol e gastrina foram correlacionados com o ângulo de Cobb e a qualidade de vida, mensurada através do questionário SRS-30.***

**Resultados:**

***Os níveos de 25-OHD estavam reduzidos em 97% dos pacientes. Observou-se uma relação inversa entre níveis de gastrina e a qualidade de vida (p=0,016). Ademais, constatou-se correlação inversa entre o valor do ângulo de Cobb e a qualidade de vida (p=0,036). Não foram observadas alterações nos níveis de cortisol. Não houve correlação do ângulo de Cobb com o nível de nenhum dos hormônios dosados.***

**Conclusão:**

*Os pacientes apresentaram níveis de 25-OHD diminuídos, fortalecendo a hipótese da sua implicação no desenvolvimento da EIA. O presente estudo também sugere que o aumento dos níveis de gastrina possa estar relacionado com pior qualidade de vida nos pacientes com EIA.* ***Nível de Evidência II, Estudo Diagnóstico.***

## INTRODUCTION

Adolescent idiopathic scoliosis (AIS) is a three-dimensional deformity of the spine with an average prevalence of 3% of the population, affecting 8 women for each man with this disorder.^1^ ^,^ ^2^ Although its exact pathogenesis has not yet been described, several authors defend a multifactorial etiology.^3^ ^,^ ^4^ Genetic changes, hormone dysfunctions, and bone mineralization deficit are among the examples of theories which have been postulated.^4^ ^,^ ^5^

Previous studies have demonstrated alterations in bone growth in AIS as well as reduced bone mineral density in these patients, but have not clarified the pathophysiology of these changes in detail.^6^ The same difficulties and uncertainties exist in relation to eating disorders and low body mass index found in patients with AIS.^7^ ^,^ ^8^

Several studies have evaluated the action of gastrin, a peptide hormone produced by G cells of the gastric antrum, in metabolism and bone quality.^9^ ^,^ ^10^ Also considering the importance of 25-hydroxyvitamin D (25-OHD) and cortisol in bone metabolism, we seek to correlate the levels of these markers with AIS, the severity of the curve, and the results obtained using the SRS-30 questionnaire.

The primary objective of this study is to evaluate the hormone profile of patients with AIS and its relation to the severity of the curve and performance on the SRS-30 questionnaire and its domains.

## MATERIALS AND METHODS

The study participants were selected from the outpatient spine clinic at the hospital where this study was conducted, read and signed the free and informed consent form. The study was approved by the Ethics Committee (CEP: 780,768). Patients of both sexes with scoliosis (Cobb angle >10° in the coronal plane in panoramic X-rays) diagnosed after 10 years of age were included. We excluded patients with any genetic syndrome, other relevant chronic diseases, or previous osteo-metabolic, muscle, or endocrinological disorders of any kind. We also excluded patients with personal history that could justify the scoliosis, such as trauma or tumors.

The Cobb angle was measured by one doctor who is an experienced spinal specialist, considering the first panoramic radiography of the spine taken after the patient began to be followed at the outpatient clinic.

We measured serum values of 25-hydroxyvitamin D (using chemical immunoassay), cortisol (using chemical immunoassay), and gastrin (using chemiluminescence).

All patients completed the SRS-30 quality of life questionnaire by the Scoliosis Research Society (SRS), which is composed of 30 questions divided into five domains (function/activity, self-image/appearance, pain, mental health, and satisfaction with management). Each question is scored from 1 (worst) to 5 (best). The maximum score possible is 150 points, and a higher score represents a better self-evaluation by the patient. (Table 1).

**Table 1:** Descriptive analysis of the quantitative variables of the study.

Variables	Mean ± SD	Median	Min-Max
Initial Cobb angle	57.65 ± 23.022	57.00	20 - 123
Quality of Life	67,00 ± 16,837	67,00	26 - 107
(SRS - 30)	67.00 ± 16.837	67.00	26 - 107
25-OHD (ng/mL)	19.40 ± 6.9	17.50	6 - 36
Gastrin (pg/mL)	35.33 ± 48.66	20.10	8 - 266
Cortisol (µg/dL)	11.98 ± 7.95	10.10	4.5 - 48.4

### Statistical methodology

The data were stored in an Excel^(r)^ for Mac spreadsheet and subsequently imported into SPSS 23^(r)^ for Mac. Continuous data were described by means and their respective standard deviations and were tested for distribution using the Kolmogorov-Smirnov test. The Pearson correlation coefficient and Spearman's rank correlation were used to analyze the correlations. Up to 5% was accepted as a type I error.

### Funding

The laboratory tests were performed in the clinical laboratory of the Instituto de Ortopedia do Hospital de Clínicas da Faculdade de Medicina da Universidade de São Paulo using this institution's resources. To measure the Cobb angle, X-rays solicited during the patients' routine care were used. No sponsorships or grants were obtained for this study or by the authors.

## RESULTS

The study was conducted with 43 people, 36 female and seven male, with an average age of 15.3 years ± 2.49.

We noted that 25-OHD levels were low in 97% of patients, classifying them as having insufficiency or deficiency. No alterations were observed in the other tests. (Table 2).

**Table 2:** Descriptive analysis of the qualitative variables of the study and reference values.

Variable	Reference values	n (%)	95% CI
Gastrin (pg/mL)			
Decreased	Below 13	(13) 30.23	15.34 - 45.12
Normal	13 - 115	(26) 60.46	44.69 - 76.24
Increased	Above 115	(2) 4.65	0.57 - 15.81
Omitted	-	(2) 4.65	0.57 - 15.81
Cortisol (µg/dL)			
Decreased	Below 5	(4) 9.30	7.61 - 34.25
Normal	5 - 25	(35) 81.39	68.60 - 94.19
Increased	Above 25	(2) 4.65	0.57 - 15.81
Omitted	-	(2) 4.65	0.57 - 15.81
25-OHD (ng/mL)			
Deficiency	Below 10	(3) 7	1.46 - 19.06
Insufficiency	10 - 30	(37) 86	74.53 - 97.57
Sufficiency	30 - 100	(1) 2.3	0.06 - 12.29
Omitted	-	-	-

An inverse correlation was noted between a low Cobb angle and the quality of life in the SRS-30 questionnaire. No significant relationships were observed between the Cobb angle and measured hormone levels. (Table 3).

**Table 3:** Relationship between Cobb angle and quantitative variables of the study.

Variables	Cobb angle	
	n	Pearson correlation	*p*
Quality of Life (SRS - 30)	43	-0.320	0.036*
25-OHD	42	0.045	0.779
	n	Spearman's rank correlation coefficient	***p***
Cortisol	41	0.274	0.083
Gastrin	41	0.152	0.342

*p*< 0.05

An inverse relationship was also noted between low gastrin values and performance on the quality of life questionnaire (SRS-30). No significant relationships were seen between the values for the other hormones and quality of life assessment. (Table 4)

**Table 4:** Relationship between SRS-30 and measured hormones.

Variables	Quality of life (SRS - 30)	
	N	Spearman's rank correlation coefficient	*p*
Gastrin	41	-0.375*	0.016*
Cortisol	41	-0.136	0.398
25OHD	42	0.193	0.220

p< 0.05

## DISCUSSION

Despite all the research about AIS, its exact pathophysiology is still unknown. From the clinical changes which were recognized and present in the patients with this disease, we attempted to evaluate their alleged causes and possible markers of severity.

Recent studies indicate an interaction between 25-OHD deficiency and development of AIS,^11^ ^,^ ^12^ which was corroborated in our study: almost all of the patients (97%) had lower 25-OHD levels than expected. Considering the small number of studies suggesting a correlation between 25-OHD and AIS, further studies are needed to support this hypothesis and to the reasons behind this variation.

No relationship was observed between vitamin D levels and Cobb angle in this study, which contradicts the single study found in the literature that correlated the two variables and showed an inverse relationship.^12^ Furthermore, no relationship was found between 25-OHD levels and quality of life assessment.

Recent studies in rats suggest that gastrin may assist in regulation of bone metabolism, and that higher gastrin levels may contribute to poorer bone quality.^9^ ^,^ ^10^ Although gastrin is present at normal levels in most patients, we found a low inverse correlation between gastrin values and the SRS-30 score, which may indicate that the most serious cases have higher serum gastrin levels. Since this is the first study correlating scoliosis and gastrin levels, further studies are required to better clarify this finding.

The cortisol levels in the patients studied were predominantly in the normal range. No relationship was observed between cortisol levels and Cobb angle or SRS-30 performance, making it unlikely they are involved in developing AIS.

Quality of life assessment in spinal diseases is common practice and is necessary for patient management. The Scoliosis Research Society provides a questionnaire for this purpose, the SRS-30.^13^ ^,^ ^14^ Some studies, however, did not find a relationship between the score on this instrument and Cobb angle, contrary to our findings in this study.

## CONCLUSION

The study participants showed low serum 25 OHD values, strengthening the hypothesis that this vitamin is involved in the development of AIS, as the current literature indicates. The present study also suggests that increased gastrin may be related to poorer quality of life in patients with AIS. No alterations were observed in the serum cortisol levels.
